# Direct vitamin k antagonist anticoagulant treatment health care costs in patients with non-valvular atrial fibrillation

**DOI:** 10.1186/1472-6963-14-46

**Published:** 2014-01-30

**Authors:** Álvaro Hidalgo-Vega, Elham Askari, Rosa Vidal, Isaac Aranda-Reneo, Almudena Gonzalez-Dominguez, Alexandra Ivanova, Gabriela Ene, Pilar Llamas

**Affiliations:** 1 Department of Economic and Financial Analysis, Faculty of Legal and Social Sciences, University of Castilla-La Mancha, Toledo, Spain; 2 Department of Hematology, Fundación Jiménez Díaz, Madrid, Spain; 3 Department of Economic and Financial Analysis, Faculty of Social Sciences, University of Castilla-La Mancha, Mancha, Avda Real Fábrica de Seda s/n, 45600 Talavera de la Reina Toledo, Spain; 4 Max Weber Institute, Majadahonda, Madrid, Spain

## Abstract

**Background:**

There is evidence suggesting that most thromboembolic complications could be prevented with adequate pharmacological anticoagulation. We estimated the direct health care costs of anticoagulant treatment with oral vitamin K antagonists in patients diagnosed with non-valvular atrial fibrillation.

**Methods:**

This observational study examined the clinical records of patients diagnosed with non-valvular atrial fibrillation who received anticoagulant treatment with oral vitamin K antagonists. Data from clinical records were used in the study: international normalized ratio, number of monitoring visits, type of anticoagulant, hospital admissions from complications, and concomitant medication. Drug cost was calculated based on the official Spanish Ministry of Health price list. Monitoring expenses were included the cost of the medical supplies used in the procedures. Hospitalization costs were calculated using the Diagnosis Related Group price for each case. Hospital visits costs were calculated by one of four different scenarios, using either the invoice rates for the regional health care authority or cost per visit as established by analytical accounting methods.

**Results:**

We collected data from 1,257 patients diagnosed with non-valvular atrial fibrillation who were receiving oral anticoagulant therapy. Depending on the scheme used, the direct health care costs for these patients ranged from €423,695 - €1,436,038 per annum. The average cost per patient varied between €392 - €1,341, depending on the approach used. Patients with international normalized ratio values within the therapeutic range on 25% of their visits represented an average cost between €441.70 - €1,592. Those within the therapeutic range on 25%–50% of visits had associated costs of €512.37 - €1,703.91. When international normalized ratio values were within the therapeutic range on 50% - 75% of the visits, the costs ranged between €400.80- €1,375.74. The average cost was €305.23 - €1,049.84 when the values were within the therapeutic range for over 75% of visits.

**Conclusions:**

Most direct health care costs associated with the sampled patients arise from the specialist-care monitoring required for the treatment. Good monitoring is inversely related to direct health care costs.

## Background

The prevalence of non-valvular atrial fibrillation (NVAF) increases with age, affecting 0.5% of the population aged 50–59 and almost 10% of those aged 80–89 [1]. The average age of patients with NVAF is 75, with a greater prevalence seen among women [1-4]. Findings from the long-term cardiovascular study in Framingham, United States (US) and the long-term cohert study in Rotterdam, the Netherlands report a one-in-four lifetime risk of developing Atrial Fibrillation (AF) for those over the age of 40 [2]. Currently, six million Europeans are diagnosed with AF, and it is estimated that this prevalence will double over the next 50 years [5]. In the US, some 2.3 million people have NVAF. Estimates based on census figures and population aging predict that this figure will rise to 3.3 million by 2020, and to 5.6 million by 2050 [6]. The most common comorbidities seen in NVAF patients include arterial hypertension, diabetes mellitus, and previous ischemic heart disease.

AF is an independent risk factor for stroke, conferring a five-fold excess risk in patients compared with those suffering from sinus rhythm. Moreover, the disease causes 10% - 15% of all ischemic strokes, and approximately one in four strokes in patients aged over 80 [3]. Therefore, given its associated stroke risk, AF has a clear and significant impact on quality of life and mortality, and is a significant risk factor for stroke recurrence [5].

There is substantial evidence suggesting that most thromboembolic complications could be prevented with adequate pharmacological anticoagulation therapy [6,7]. Long-term oral anticoagulation therapy (OAT) with vitamin K antagonists (VKAs) is prescribed as prophylaxis against strokes and other embolic events in patients with AF or mechanical heart valves. VKAs such as warfarin were the cornerstone of pharmacotherapy for AF in patients with a moderate to high thromboembolic risk [8]. In clinical trials, anticoagulation with adjusted-dose warfarin has been shown to reduce the risk of ischemic stroke in NVAF patients by one-half to two-thirds. For this reason, evidence-based clinical guidelines recommend anticoagulation with warfarin for patients with NVAF who bear a moderate to high risk of stroke: 90% of the NVAF population [9]. It has been shown that improved anticoagulant control can be achieved through frequent monitoring of the international normalized ratio (INR), resulting in improved health outcomes. However, adherence to warfarin is problematic and strongly associated with poor anticoagulation control during all phases of therapy. Hence, addressing correct warfarin dosage and poor adherence issues hold significant promise for improving its use as one of the most commonly prescribed drugs available.

Most NVAF patient interventions and hospital admissions are as a result of the treatment and management of the disease. Twenty to thirty percent of AF patients who receive oral VKAs are hospitalized at some point in their life [10-13]. In the US, NVAF causes approximately 350,000 hospital admissions per year, costing approximately $3 billion USD [14]. In Europe, direct health care costs per patient varied between €1,507- €2,328 annually depending on the country. Of those costs, 40% were attributable to hospital admissions, 30% to interventions performed as a result of the disease, and 10% to drug therapy [5]. Research conducted in the UK that examined patients receiving warfarin treatment showed that 34% of the costs paid by the National Health Service (NHS) were spent on INR monitoring [15].

Despite the significant number of AF cost estimation studies, only two have focused on Spain [16]. Of these two studies, one used data from four hospitals where 47% of the sample had AF. The other patients in the sample were undergoing OAT but had a different underlying condition (such as mechanical heart prostheses or rheumatic heart disease) and hence the results were disaggregated [17]. The other study was conducted in five European countries (including Spain) and estimated AF costs. However, the patients included in the study were receiving antithrombotic therapy rather than OAT [5].

The objective of this study was to estimate the direct health care costs of patients with NVAF who receive chronic OAT with VKAs from the perspective of a specialized anticoagulation unit.

## Methods

The study used an observational approach. A retrospective analysis was performed using the clinical records of patients who had visited the Hematology Service of the Hospital Fundación Jiménez Díaz, Madrid, Spain between October 1, 2009 and September 30, 2010. The databases used did not contain any variable allowing the individual identification of patients. According to the legislation in Spain, the characteristics (observational methodology and retrospective analysis) of this study do not make it necessary to obtain authorisation from the hospital Ethics Committee [18].

### Patient selection

Patient data was extracted from anonymous clinical records stored in the hospital databases. Subjects were selected by members of the research team who worked independently from the group responsible for statistical analysis. Patients with diagnosed NVAF and receiving OAT (warfarin sodium or acenocoumarol) were eligible for inclusion. There was no gender or age discrimination. To avoid including data from patients who regularly attend a different hospital, the team only included data from patients that continuously visited the hospital for a minimum period of five months and one day when estimating the patient costs.

### Data collection

The data collected included the number of visits made by each patient, the INR value at the time of each visit, the OAT dose and regime (measured in average weekly dose per patient, and type of drug), concomitant medication (measured in average weekly dose per patient), the devices required for INR monitoring (a test strip, a pipette, and a lancet), and hospital admissions caused by complications resulting from INR values outside the therapeutic range (TR). Where the patient’s INR was greater than or equal to five, the result was confirmed by testing either capillary blood or venous blood.

### Data analysis

A previously developed model was used to measure the period of time when patient INR values fell within the TR [19]. Visits were classified as either “visits within the TR” (1.9 < INR < 3.1) or “visits outside the TR” (INR < 1.9 or INR > 3.1). To calculate the time within the therapeutic range (TTR), we estimated the number of days when each patient exhibited values within the therapeutic range and recorded the INR value of each of these days. The INR value recorded on a given visit cannot be taken in isolation. Clinical experience shows that INR readings fluctuate between visits, and values can fall within the TR at some times but not at others. Several further adjustments were made to avoid error. Patients who had only visited the hospital once were excluded from the study. Where more than 56 days had elapsed between any two visits the data were considered not linear and, therefore, these periods were excluded from the calculation as previously recommended [19]. INR data collected in the five days following a known interruption of treatment were considered void.

### Health-care resources assessment

The direct health care costs [20] were calculated as the sum of the following costs: OAT using pharmacological products, INR measurements, medical visits, rescue drugs (heparin/vitamin K), and hospitalizations. We consulted several sources to express the health care resources consumed in monetary terms. Drug prices (OAT using pharmacological products and rescue drugs) were obtained from the Spanish official drug database [21]. The annual average treatment cost per-patient was calculated using the following formula:

$$\mathrm{CAP}=\sum \left(\mathrm{DD}\times P\times \mathrm{DT}\right)$$

Where *DD* represents daily dose mg/day, *P* represents price per mg, and *TD* represents treatment days.

Two different methods were used to calculate the cost of hospital visits. The first included the total costs incurred by the hospital’s hematology service (such as IT resources, cleaning, electricity, water and other overheads) in addition to the costs of the healthcare personnel who intervened during the visit (physicians, nurses, and nurse assistants). The second method accounted for hospital visit costs by applying the 2010 regional health service price list as published in the official region gazette [22]. Four healthcare resource use scenarios were established. The regional health service price was applied for all visits in scenario 1. In scenario 2, the regional health service price was applied for the first and subsequent visits and different prices were imputed for visits with and without complications. In the third scenario, the first visit was valued at the regional health service price, and subsequent visits were valued at the price obtained from the hospital cost accounting records. Finally, scenario 4 assessed every hospital visit at the price obtained from the hospital cost accounting records.

INR measurements included internal assessments of the health care resources required to perform the test and, where necessary, the medical equipment needed to confirm the results obtained (measurements in capillary or venous blood). Hospital admissions were measured according to the diagnosis-related group (DRG) price applied for each particular admission (admissions because of hemorrhage or thrombosis). All costs were estimated in current euros for the year 2010.

## Results

The final survey size was 1,257 patients ,with a mean age of 78.41 (standard deviation (SD) = 9.11). Of the patients, 17% (n = 208) had received an NVAF diagnosis for the first time. The health care resource assessment used data from those who had regularly visited the hospital for a period of over five months and one day (n = 944; 75% of the total population). Table 1 shows a breakdown of both patient samples.

**Table 1:** Breakdown of patients included in the study

	**Male**		**Female**		**Overall**	
	**Average %**	**SD**	**Average %**	**SD**	**Average %**	**SD**
**Population**	**N = 581**		**N = 676**		**N = 1,257**	
**Age**	76.55	9.47	80.01	8.48	78.41	9.11
**Age groups**						
27–74 years	33.22		19.82		26.01	
75–80 years	29.78		26.92		28.24	
81–84 years	17.90		24.11		21.20	
85–99 years	19.10		29.14		24.50	
Visits*	13.93	6.58	14.22	6.51	14.08	6.54
Percentage of visits with incident*	19.11	18.93	19.35	18.53	19.24	18.71
**INR**	2.50	0.40	2.53	0.36	2.52	0.38
**Average weekly dose of VKA (mg)**	12.93	6.46	11.99	5.96	12.43	6.21
**Patients participating in the study for over 5 months**	N = 435		N = 509		N = 944	
**Age**	76.37	9.27	79.41	8.32	78.01	8.90
**Age groups**						
27–74 years	34.94		22.40		28.18	
75–80 years	29.43		28.49		28.92	
81–84 years	19.31		22.59		21.08	
85–99 years	16.32		26.52		21.82	
Visits*	16.59	5.03	16.78	4.98	16.69	5.00
Percentage of visits with incident*	15.88	13.71	17.17	14.24	16.58	14.01
**INR**	2.50	0.25	2.53	0.25	2.52	0.25
**Average weekly dose of VKA (mg)**	12.83	6.55	12.05	5.96	12.41	6.25

Note: Stars denote statistically significant differences between the sample and the population at 95% confidence.

**Figure 1:**
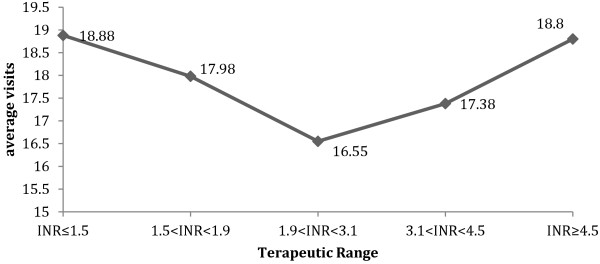
**Average visits by therapeutic range.** Sample: Total visits (17,704).

A total of 17,704 visits were recorded. 69.7% of the surveyed patients made 11 - 20 visits during the study period. The average monthly number of visits per patient was 1.17. Of all visits, 61.8% were classified as having INR values “within the TR”. In the remaining 38.2% of visits, the INR value fell outside the TR, and included more patients with abnormally low INR values (INR < 1.9; number of visits = 3,203) than those with abnormally high INR values (INR > 3.1; number of visits = 2,817). Figure 1 illustrates the number of visits, by INR values, for all visits made by the 1,257 patients.

52.44% of patients exhibited an INR value below the TR at some stage during the study period (INR < 1.5), and 31.14% of patients had an INR reading above the TR at least once (INR > 4.5). Only 2.54% of the patients had completely stable INR values throughout the study period. The prices for anticoagulant treatments and all the unit costs included in the estimated direct health care costs appear in Table 2.

**Table 2:** Unit costs used in the study

**Health-care resource**	**Scenario 1**	**Scenario 2**	**Scenario 3**	**Scenario 4**
First-hospital visit ^a,c^	€124	€124	€124	€17.41
Second and subsequent hospital visits ^a,c^	€74	With incident: €74	€17.41	€17.41
		Without incident: €17.41		
Anticoagulant treatment (VKA)^b^	€0.098/mg			
Rescue medication^b^	LMWH €3.121/dose			
	Vitamin K: €0.376			
INR monitoring^c^	€2.73			
DRG hospital admission^c^	Thrombosis: €5,418			
	Hemorrhage: €3,736			

^a^Source: regional health service price list (reference [22]).

^b^Source: Reference [21].

^c^Source: hospital cost accounting.

*LMWH* low molecular weight heparin.

The average weekly anticoagulant treatment dose was 12.41 mg (SD = 6.25). The total annual cost of the anticoagulant treatment was €16,546.47, with an average annual per-patient cost of €17.53 (SD = 9.01). No statistically significant differences were observed based on gender (p = 0.081). However, the cost of INR monitoring decreased in conjunction with increasing patient age (p = 0.00). The total cost of INR monitoring was €43,751.36, with an annual per-patient cost of €46.35 (SD = 14.51). No statistically significant differences were observed based on gender (p = 0.592) or age (p = 0.995). The total annual cost of medical visits were estimated at €1,169,492 for scenario 1, €438,352 for scenario 2, €281,530 for scenario 3, and €278,282 for scenario 4. The mean cost per patient varied from €1,238.87 (SD = 370.31) in the first scenario to €290.55 (SD = 87) in the fourth scenario. No gender-based differences were detected (p = 0.988, p = 0.340, p = 0.955, and p = 0.990 respectively). Throughout the study period, nine patients were hospitalized from complications linked to the anticoagulant therapy. All of these patients were over 65 years of age, five were admitted to hospital following a thrombotic episode, and four following a hemorrhage. The total cost of hospital admissions for these nine patients was €32,880, with an average per-patient cost of €3,653.33. The cost of rescue medication was €3,439.04, with a mean annual cost per patient of €3.64 (SD: 5.53).

**Figure 2:**
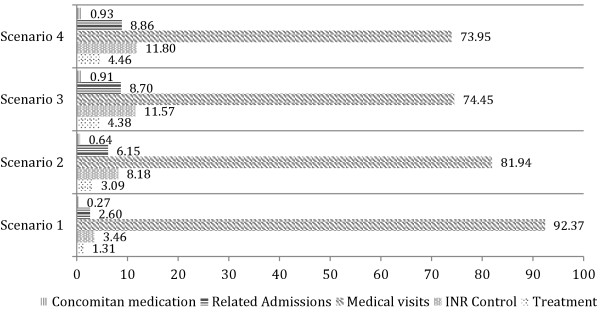
**Percentage breakdown of overall costs including visits and cost sub-groupings.** Source: Compiled by the authors (n = 944).

To compare each cost item, examine the example of scenario 4 in Figure 2. Here the cost of rescue medication represents 0.93% of the total cost, OAT 4.46%, related admission 8.86%, INR control 11.80%, and medical visits 73.95%. It is clear that across all scenarios the item representing the largest proportion of the total is that of hospital visits: 73.95% in scenario 4 , 74.45% in scenario 3, 81.94% in scenario 2, and 92.37% in scenario 1 (see Figure 2).

The Rosendaal method [19] revealed that the longer patients remained within the TR, the lower was the cost per patient (p = 0.00). Therefore, as shown in Table 3, under scenario 4, the patients in Group 2 incurred an average per-patient cost of €570.29. This sum decreased to €421 in Group 3 and €340.92 in Group 4. Thus, the difference in cost between Group 2 and Group 4 is 40.22% under scenario 4 and 52.74% under scenario 1.

**Table 3:** Average per-patient cost based on time within the TR (in 2010 Euros)

	**Scenario 1**	**Scenario 2**	**Scenario 3**	**Scenario 4**
Group 1: Less than 25% of time in TR	694.04	382.77	198.84	198.84
Group 2: 25% - 50% of time in TR	1,771.97	911.32	591.08	570.29
Group 3: 50% - 75% of time in TR	1,478.72	633.78	429.31	421.00
Group 4: Over 75% of time in TR	1,160.09	453.92	345.88	340.92
Total	1,341.22	566.70	400.58	392.90

Source: Compiled by the authors. Baseline: Patients included in the sample for a minimum of five months and one day (n = 944).

Non-standard format.

Table 4 lists the total average costs and their respective per-patient and per-year cost items for two groups of scenario 1 patients: those with TTR under 50% and those whose TTR was equal to or greater than 50%. The mean costs for patients whose TTR was under 50% was €553, while those patients whose TTR was 50% or over was €376. This represents a difference of €176, or over 44% of the average total cost. The three cost components underlying this difference are medical visits, hospital admissions and concomitant medication. These three cost items account for €160 of the €176 difference seen between the two groups. When analyzing the cost difference between the two groups in terms of a percentage of the overall cost, the hospital admissions and the costs of concomitant medication for patients with TTR below 50% amount to 218.68% of the average cost of hospital admissions and 226.21% of the average cost of concomitant medication. Text for this sub-section.

**Table 4:** Average cost according to TTR, type of cost (in Euros), and percentages

	**Total**	**Treatment**	**INR control**	**Medical visits**	**Related admissions**	**Concomitanmedication**
TTR < 49	553.01	20.94	58.13	356.62	10.88	106.44
TTR ≥50	376.85	17.19	45.17	283.93	2.92	27.65
Total	392.90	17.53	46.35	290.55	3.64	34.83
Difference (Dif)	176.16	3.75	12.96	72.69	7.96	78.79
% difference relative to TC	44.84%	21.39%	27.96%	25.02%	218.68%	226.21%

Source: Compiled by the authors. Baseline: Patients included in the sample for a minimum of five months and one day (n = 944).

## Discussion

The characteristics of the patients included in this study differ slightly from those of the AF patients included in the PREV-ICTUS study, where there was a mean age of 75.0 ± 7 and 49.5% were female. Moreover, in the PREV-ICTUS study only 33% of the AF patients were receiving OAT [23]. The CARDIOTENS 1999 study was broader and included AF patients with a mean age of 68.4 ± 10.3 years, 55.9% female participants, and only 28% receiving OAT [24]. The sampling differences between the current study and previous studies (particularly the proportion of females) may be because this study only included patients with NVAF who were also receiving VKA treatment. However, a review of investigations carried out in patients with NVAF receiving VKA treatment reveals that our population is very similar to those of other studies, as can be seen in a recent work which used a similar population and was made up of 56% women and had an average age of 73 ± 8 years [25]. A study covering several countries, including Spain, examined patients with NVAF who were undergoing VKA treatment where the average age of the patients was 71.6 years and 56% were male [26].

The average annual number of hospital visits made by the subjects in the study was 14.08 ± 6.54. This figure was slightly higher than that of other studies carried out in Spain to date. One study found the average number of visits to be 12.1 [27], although it was conducted over a period of ten years in a rural area. A further study, undertaken in 2003 and included 20,347 patients receiving VKA treatment monitored by four anticoagulation units within large hospitals in Spain, found that the average annual number of visits per patient was 10.42 [17]. Of the four centers, the one that received the lowest number of visits recorded an average of 8.21 visits/person/year, while the one with the highest number of visits recorded an average of 15.70. Hence, the figures recorded in our study can be considered representative of clinical practice in Spain.

The admission rates included in our work (admission from hemorrhage at 0.31/100 patients/year, and from pulmonary embolism at 0.39/100 patients/year) are near average, compared to the results of the aforementioned study carried out in four large hospitals that reported admissions from hemorrhage at 0.88/100 patients/year and from pulmonary embolism at 0.62/100 patients/year [17]. In the ten-year follow-up study performed in a cohort of rural patients, the rates were 1.24/100 patients/year for hemorrhage and 0.21/100 patients/year for thrombosis [27].

One study performed in four large Spanish National Health Service hospitals [17] found higher costs per treatment higher than ours, the difference solely because of variations in dose, as the price of acenocoumarol is the same in both cases, €0.03/mg. INR monitoring costs include medical supplies, logged expenses, and medical personnel. Our results can be compared with this study by subtracting the cost of medical staff, which can be analyzed separately. Our study includes the clinical visits costs needed for repeated INR monitoring when the associated values are over 4.5. This can explain why all visits yielding results above 4.5 reflect two INR monitoring sessions rather than one.

Our methodology and the data obtained, allow us to accurately estimate the actual cost associated with such patients for the Spanish National Health System. To our knowledge, this is the first study of its kind to be published.

## Conclusions

The study results demonstrate that patients receiving VKA treatment require exhaustive follow-up, thus making anticoagulant therapy a costly intervention. The need for dose adjustments are brought on by numerous external but nonetheless common factors, such as changes in diet, visits to the dentist, or vacation periods. Thus, the National Health Service is required to provide specific monitoring and dosage control services in addition to personalized clinical observation, thereby increasing the cost of the treatment. Some studies support the creation of educational initiatives to improve the adherence to and proper monitoring of anticoagulant therapy. With the gradual introduction of new drugs, new approaches will arise in the clinical oversight of this pharmacological treatment. Our results provide updated and more accurate data than previously available. Our results provide information that is relevant to decision-making and that might prove necessary for future cost-effectiveness studies comparing current oral anticoagulation drugs with newly developed drugs. Up to date, close observation of therapeutic ranges by specialist services represents the best way to contain the health care costs associated with treating NVAF patients receiving VKA treatment.

## Competing interests

The Max Weber Institute recived a non-oriented grant from Boehringer Ingelheim Spain for funding parts of this study (collecting data, translation services and publication fees). AGD and AI are employees of Max Weber Institute. However, in no case did this situation influence the results presented. All others authors declare that they have no competing interests.

## Authors’ contributions

AH conceived and designed the research, drafted the manuscript and critically reviewed the manuscript for important intellectual content. EA, RV and IA analyzed and interpreted the data and drafted the manuscript. AG and AI acquired the data and performed statistical analysis. PL critically reviewed the manuscript for important intellectual content. All authors read and approved the final manuscript.

## Pre-publication history

The pre-publication history for this paper can be accessed here:

http://www.biomedcentral.com/1472-6963/14/46/prepub

## References

[B1] Candel FJ Matesanz M Cogolludo F Candel I Mora C Bescos T Martín M Vila i Costa I Prevalence of atrial fibrillation and relationed factors in a population in the centre Madrid An Med Interna 2004 14 10 477 482 15511197 10.4321/s0212-71992004001000003

[B2] Charlemagne A Blacher J Cohen A Collet JP Dievart F de Groote P Hanon O Leenhardt A Pinel JF Pisica-Donose G Epidemiology of atrial fibrillation in France: extrapolation of international epidemiological data to France and analysis of French hospitalization data Arch Cardiovasc Dis 2011 14 2 115 124 10.1016/j.acvd.2010.11.012 21402346

[B3] Feinberg WM Blackshear JL Laupacis A Kronmal R Hart RG Prevalence, age distribution, and gender of patients with atrial fibrillation. Analysis and implications Arch Intern Med 1995 14 5 469 473 10.1001/archinte.1995.00430050045005 7864703

[B4] Go AS Hylek EM Phillips KA Chang Y Henault LE Selby JV Singer DE Prevalence of diagnosed atrial fibrillation in adults: national implications for rhythm management and stroke prevention: the AnTicoagulation and Risk Factors in Atrial Fibrillation (ATRIA) Study JAMA 2001 14 18 2370 2375 10.1001/jama.285.18.2370 11343485

[B5] Ringborg A Nieuwlaat R Lindgren P Jonsson B Fidan D Maggioni AP Lopez-Sendon J Stepinska J Cokkinos DV Crijns HJ Costs of atrial fibrillation in five European countries: results from the Euro Heart Survey on atrial fibrillation Europace 2008 14 4 403 411 10.1093/europace/eun048 18326853

[B6] Gersh BJ Tsang TSM Seward JB The changing epidemiology and natural history of nonvalvular atrial fibrillation: clinical implications Trans Am Clin Climatol Assoc 2004 14 149 17060964 PMC2263785

[B7] Fuster V Rydénn LE Cannom DS Crijns HJ Curtis AB Ellenbogen KA Halperin JL Heuzey J-YL Neal Kay G Lowe JE ACC/AHA/ESC: Guía de práctica clínica 2006 para el manejo de pacientes con fibrilación auricular. Versión resumida Rev Esp Cardiol 2006 14 12 1328 1328 17194429

[B8] Lip GYH Edwards SJ Stroke prevention with aspirin, warfarin and ximelagatran in patients with non-valvular atrial fibrillation: a systematic review and meta-analysis Thromb Res 2006 14 3 321 333 10.1016/j.thromres.2005.08.007 16198396

[B9] Bushnell CD Matchar DB Pharmacoeconomics of atrial fibrillation and stroke prevention Am J Manag Care 2004 14 3 Suppl S66 S71 15152748

[B10] Davy JM Roubille F Tri Cung T Massin F Crausac F Raczka F Pasquie JL Atrial fibrillation in 2010: an increasing morbidity and mortality burden Ann Cardiol Angeiol 2010 14 Suppl 1 S4 S13 10.1016/S0003-3928(10)70002-0 21211625

[B11] Hylek EM Go AS Chang Y Jensvold NG Henault LE Selby JV Singer DE Effect of intensity of oral anticoagulation on stroke severity and mortality in atrial fibrillation N Engl J Med 2003 14 11 1019 1026 10.1056/NEJMoa022913 12968085

[B12] Kim MH Lin J Hussein M Kreilick C Battleman D Cost of atrial fibrillation in United States managed care organizations Adv Ther 2009 14 9 847 857 10.1007/s12325-009-0066-x 19768638

[B13] Wu EQ Birnbaum HG Mareva M Tuttle E Castor AR Jackman W Ruskin J Economic burden and co-morbidities of atrial fibrillation in a privately insured population Curr Med Res Opin 2005 14 10 1693 1699 10.1185/030079905X65475 16238910

[B14] Coyne KS Paramore C Grandy S Mercader M Reynolds M Zimetbaum P Assessing the direct costs of treating nonvalvular atrial fibrillation in the United States Value Health 2006 14 5 348 356 10.1111/j.1524-4733.2006.00124.x 16961553

[B15] Abdelhafiz AH Wheeldon NM Use of resources and cost implications of stroke prophylaxis with warfarin for patients with nonvalvular atrial fibrillation Am J Geriatr Pharmacother 2003 14 2 53 60 10.1016/S1543-5946(03)90001-8 15555467

[B16] Wolowacz SE Samuel M Brennan VK Jasso-Mosqueda JG Van Gelder IC The cost of illness of atrial fibrillation: a systematic review of the recent literature Europace 2011 14 10 1375 1385 10.1093/europace/eur194 21757483

[B17] Navarro JL César JM Fernández MA Fontcuberta J Reverter JC Gol-Freixa J Tratamiento anticoagulante oral. Estudio Coste-Beneficio Rev Adm Sanit 2008 14 3 525 542

[B18] Ministry of Health Social Services and Equality Orden SAS/3470/2009, de 16 de diciembre, por la que se publican las directrices sobre estudios posautorización de tipo observacional para medicamentos de uso humano, Volume SAS/3470/2009 2009 Madrid: Spanish Govermment

[B19] Rosendaal FR Cannegieter SC van der Meer FJ Briet E A method to determine the optimal intensity of oral anticoagulant therapy Thromb Haemost 1993 14 3 236 239 8470047

[B20] Drummond M O’Brien B Stoddart G Method for the economic evaluation of health care programmes 1997 Oxford: Oxford University Press

[B21] Ministerio de Sanidad, Política Social e Igualdad Nomenclator DIGITALIS-INTEGRA [http://www.msssi.gob.es/profesionales/nomenclator.do]

[B22] Madrid Official Regional Gazette 2010 [http://www.bocm.es/bocm/Satellite?language=es&pagename=Boletin%2FPage%2FBOCM_home]

[B23] Cea-Calvo L Redon J Lozano JV Fernandez-Perez C Marti-Canales JC Llisterri JL Gonzalez-Esteban J Aznar J Prevalence of atrial fibrillation in the Spanish population aged 60 years or more. The PREV-ICTUS study Rev Esp Cardiol 2007 14 6 616 624 10.1157/13107118 17580050

[B24] García-Acuña JM González-Juanatey JR Alegría Ezquerra E González Maqueda I Listerri JL La fibrilaciónn auricular permanente en las enfermedades cardiovasculares en España. Estudio CARDIOTENS 1999 Rev Esp Cardiol 2002 14 09 943 952 10.1016/S0300-8932(02)76733-X 12236924

[B25] Ruiz Ortiz M Romo E Mesa D Delgado M Anguita M Castillo JC Arizon JM Suarez de Lezo J Oral anticoagulation in nonvalvular atrial fibrillation in clinical practice: impact of CHADS(2) score on outcome Cardiology 2010 14 3 200 204 10.1159/000284450 20160440

[B26] Pengo V Pegoraro C Cucchini U Iliceto S Worldwide management of oral anticoagulant therapy: the ISAM study J Thromb Thrombolysis 2006 14 1 73 77 10.1007/s11239-006-5580-y 16475046

[B27] Trullas-Vila JC Bisbe-Company J Freitas-Ramirez A Soler-Simon S Bisbe-Company V Roncero-Vidal JM Gispert-Magarolas R Ten-year experience with acenocoumarol treatment in an ambulatory cohort of Spanish patients J Thromb Thrombolysis 2009 14 4 436 443 10.1007/s11239-009-0311-9 19225864

